# Supplementary material for: Tissue inflammation induced by constitutively active STING is mediated by enhanced TNF signaling
Source: eLife. 2025 Mar 20;14:e101350. doi: 10.7554/eLife.101350 (PMC11996172; doi:10.7554/eLife.101350)
Supplement: Supplementary file 1. — The following antibodies were used for FACS analysis. [file elife-101350-supp1.docx]

**Supplemental Table S1. List of antibodies for FACS analysis (all from BioLegend)**

| **Antibody** | **Dilution** | **Cat no** |
| --- | --- | --- |
| Anti-CD45.2 | 1:300 | 109831 |
| Anti-CD3 | 1:1000 | 100306 |
| Anti-CD4 | 1:1000 | 100449 |
| Anti-CD8 | 1:1000 | 140415 |
| Anti-CD62L | 1:1000 | 104411 |
| Anti-CD44 | 1:1000 | 103027 |
| Anti-CD19 | 1:1000 | 115545 |
| Anti-CD11b | 1:1000 | 101215 |
| Anti-Ly-6C | 1:1000 | 128025 |
| Anti-CD25 | 1:1000 | 101909 |
