# Supplementary material for: Tissue inflammation induced by constitutively active STING is mediated by enhanced TNF signaling
Source: eLife. 2025 Mar 20;14:e101350. doi: 10.7554/eLife.101350 (PMC11996172; doi:10.7554/eLife.101350)
Supplement: Supplementary file 2. — The following antibodies were used for staining of brain sections. [file elife-101350-supp2.docx]

**Supplemental Table S2. List of antibodies for immunofluorescence staining of brain sections**

| **Antibody** | **Dilution** | **Source** | **Cat. no.** |
| --- | --- | --- | --- |
| Anti-Tyrosine Hydroxylase, sheep | 1:2000 | Pel-Freez | P60101 |
| Anti-GFAP, chicken | 1:1000 | Abcam | ab4674 |
| Anti-Iba1, guinea pig | 1:2000 | Histo Sure | HS-234308 |
| Alexa 488 conjugated donkey anti-sheep | 1:1000 | Invitrogen | A11015 |
| Alexa 647 conjugated donkey anti-chicken | 1:500 | Jackson ImmunoResearch | 703-605-155 |
| CF 555 conjugated donkey anti-guinea pig | 1:1000 | Sigma-Aldrich | SAB4600298 |
| Hoechst | 1:2000 | Invitrogen | H3570 |
