# Supplementary material for: Tissue inflammation induced by constitutively active STING is mediated by enhanced TNF signaling
Source: eLife. 2025 Mar 20;14:e101350. doi: 10.7554/eLife.101350 (PMC11996172; doi:10.7554/eLife.101350)
Supplement: Supplementary file 3. — The following primers were used for qRT-PCR analysis. [file elife-101350-supp3.docx]

**Supplemental Table S3. List of qRT-PCR primers**

| **Gene** | **Primer forward (5’- 3’)** | **Primer reverse (5’- 3’)** |
| --- | --- | --- |
| *Cxcl10* | AACTGACTGCTCGCAATAATGT | GTAACACAGCAATGCCTCTTGT |
| *Mx1* | AACCCTGCTACCTTTCAA | AAGCATCGTTTTCTCTATTTC |
| *Sting1* | CTGCTGACATATACCTCAGTTG | GAGCATGTTGTTATGTAGCTG |
| *Tnf* | CCTGTAGCCCACGTCGTAG | GGGAGTAGACAAGGTACAACCC |
| *Il1b* | GAAATGCCACCTTTTGACAGTG | TGGATGCTCTCATCAGGACAG |
| *Hprt1* | TCAGTCAACGGGGGACATAAA | GGGGCTGTACTGCTTAACCAG |
| *Rpl13a* | AGCCTACCAGAAAGTTTGCTTAC | GCTTCTTCTTCCGATAGTGCATC |
| *Eef2* | CCGACTCCCTTGTGTGCAA | AGTTCAGGTCGTTCTCAGAGAG |
