# Supplementary material for: Tissue inflammation induced by constitutively active STING is mediated by enhanced TNF signaling
Source: eLife. 2025 Mar 20;14:e101350. doi: 10.7554/eLife.101350 (PMC11996172; doi:10.7554/eLife.101350)
Supplement: Supplementary file 4. — Analysis of effect size and power were performed by G*Power 3.1.9.4, effect size d convention (<0.5 = small, 0.5–0.8=medium, >0.8 = large effect), and effect size f convention (<0.25 = small, 0.25–0.4=medium, >0.4 = large effect). [file elife-101350-supp4.docx]

**Supplemental Table S4. List of effect size and power calculations**

| **Figure** | **Effect size** | | | **Power** |
| --- | --- | --- | --- | --- |
|  | **Cohen's d** | **Cohen's f** | **convention** |  |
| **1A** | n.d. | 0.261 | medium | 0.242 |
| **1C** | n.d. | 0.601 | large | 0.928 |
| **1D** | n.d. | 0.640 | large | 0.956 |
| **1E** | n.d. | 0.604 | large | 0.930 |
| **1F** | n.d. | 0.583 | large | 0.911 |
| **1G** | n.d. | 0.303 | medium | 0.358 |
| **1H** | n.d. | 0.681 | large | 0.975 |
| **1I** | n.d. | 0.413 | large | 0.617 |
| **1J** | n.d. | 0.683 | large | 0.976 |
| **2A** | n.d. | 0.362 | medium | 0.459 |
| **2B** | n.d. | 0.440 | large | 0.638 |
| **2C** | n.d. | 0.299 | medium | 0.323 |
| **2D** | n.d. | 1.016 | large | 0.999 |
| **2E** | n.d. | 0.464 | medium | 0.689 |
| **2F** | n.d. | 0.888 | large | 0.999 |
| **2G** | n.d. | 0.858 | large | 0.998 |
| **2H** | n.d. | 0.958 | large | 0.999 |
| **2I** | n.d. | 0.978 | large | 0.999 |
| **2J** | n.d. | 0.993 | large | 0.999 |
| **2K** | n.d. | 0.657 | large | 0.916 |
| **2L** | n.d. | 0.312 | medium | 0.309 |
| **2M** | n.d. | 0.966 | large | 0.999 |
| **2N** | n.d. | 0.948 | large | 0.999 |
| **3B** | 0.969 | n.d. | large | 0.356 |
| **3C** | 0.341 | n.d. | small | 0.086 |
| **3D** | 1.384 | n.d. | large | 0.621 |
| **4A** | n.d. | 0.483 | large | 0.749 |
| **4B** | n.d. | 0.693 | large | 0.974 |
| **4C** | n.d. | 0.798 | large | 0.995 |
| **4D** | n.d. | 0.679 | large | 0.969 |
| **4E** | n.d. | 1.117 | large | 0.999 |
| **4F** | n.d. | 0.859 | large | 0.999 |
| **4H** | n.d. | 3.299 | large | 1.000 |
| **5D-Medium** | 0.669 | n.d. | medium | 0.719 |
| **5D-TNF** | 0.964 | n.d. | large | 0.999 |
| **5D-LPS** | 1.256 | n.d. | large | 0.999 |
| **5D-LPS-TEM** | 0.724 | n.d. | medium | 0.909 |
| **5E-Medium** | 0.785 | n.d. | medium | 0.848 |
| **5E-TNF** | 2.296 | n.d. | large | 1.000 |
| **5E-LPS** | 0.441 | n.d. | small | 0.539 |
| **5E-LPS-TEM** | 0.361 | n.d. | small | 0.391 |
| **5F-Medium** | 0.500 | n.d. | small | 0.478 |
| **5F-TNF** | 0.659 | n.d. | medium | 0.830 |
| **5F-LPS** | 0.590 | n.d. | medium | 0.741 |
| **5F-LPS-TEM** | 0.220 | n.d. | small | 0.164 |
| **5G-Medium** | 0.000 | n.d. | non | 0.050 |
| **5G-TNF** | 1.115 | n.d. | large | 0.998 |
| **5G-LPS** | 0.352 | n.d. | medium | 0.397 |
| **5G-LPS-TEM** | 0.338 | n.d. | medium | 0.371 |
| **S1A** | n.d. | 0.167 | small | 0.132 |
| **S1C** | n.d. | 0.611 | large | 0.936 |
| **S1D** | n.d. | 0.617 | large | 0.941 |
| **S1E** | n.d. | 0.290 | medium | 0.330 |
| **S1F** | n.d. | 0.232 | small | 0.220 |
| **S1G** | n.d. | 0.609 | large | 0.935 |
| **S1H** | n.d. | 1.062 | large | 0.999 |
| **S1I** | n.d. | 0.392 | medium | 0.568 |
| **S1J** | n.d. | 0.216 | small | 0.194 |
| **S2A** | n.d. | 0.502 | large | 0.742 |
| **S2B** | n.d. | 0.711 | large | 0.969 |
| **S2C** | n.d. | 0.478 | large | 0.695 |
| **S2D** | n.d. | 0.107 | small | 0.077 |
| **S2E** | n.d. | 0.226 | small | 0.188 |
| **S2F** | n.d. | 0.566 | large | 0.847 |
| **S2G** | n.d. | 0.712 | large | 0.975 |
| **S2H** | n.d. | 0.399 | medium | 0.545 |
| **S2I** | n.d. | 0.833 | large | 0.996 |
| **S2J** | n.d. | 0.477 | large | 0.755 |
| **S2K** | n.d. | 0.685 | large | 0.976 |
| **S2L** | n.d. | 0.666 | large | 0.969 |
| **S2M** | n.d. | 0.518 | large | 0.828 |
| **S2N** | n.d. | 0.315 | medium | 0.385 |
| **S4A** | n.d. | 0.177 | medium | 0.134 |
| **S4B** | n.d. | 0.316 | medium | 0.358 |
| **S4C** | n.d. | 0.402 | large | 0.551 |
| **S4D** | n.d. | 0.400 | large | 0.547 |
| **S4E** | n.d. | 0.572 | large | 0.900 |
| **S4F** | n.d. | 0.142 | small | 0.107 |
| **S4G** | n.d. | 0.545 | large | 0.835 |
| **S4H** | n.d. | 0.255 | medium | 0.242 |
| **S4I** | n.d. | 0.395 | medium | 0.536 |
| **S4J** | n.d. | 0.194 | small | 0.154 |
| **S4K** | n.d. | 0.573 | large | 0.873 |
| **S4L** | n.d. | 0.269 | medium | 0.267 |
| **S4M** | n.d. | 0.610 | large | 0.913 |
| **S4N** | n.d. | 0.675 | large | 0.960 |
| **S4O** | n.d. | 0.579 | large | 0.880 |
